# 

**Published:** 1912-10-12

**Authors:** 


### THE NEWSVENDORS' ANNUAL DINNER.

On November 4 the anniversary dinner of the Newsvendors' Benevolent and Provident Institution will be held at De Keyser's Hotel, when Sir Frank Newnes, Bart., will preside. In commemoration of Charles Dickens' influence as President from 1854 to 1870, it is desired to establish a number of pensions. £2,000 would provide for two men's and one woman's pension in perpetuity.

### Appointments.

The following gentlemen have been appointed resident medical officers to the Royal Southern Hospital for the ensuing six months: Robert Findlay, M.B., Ch.B., Glasgow, house physician; Hugh Pierce, M.B., B.Ch., M.R.C.S., L.R.C.P., house physician; T. H. Martin, M.B., Ch.B., Liverpool, house surgeon; W. Hilton Parry, M.B., Ch.B., Liverpool, house surgeon; N. G. Chavasse, M.A., M.B., B.Ch., M.R.C.S., L.R.C.P., house surgeon.

### BUREAU OF INFORMATION.

### Rules for Correspondents.

1. Every letter, on whatever subject, **must be accompanied by the coupon** to be cut from the back cover (inside page) of The Hospital, current issue, and must contain the name and address of the correspondent with pseudonym for publication if desired.

2. Replies by post cannot be given, save under exceptional circumstances at the Editor's discretion.

3. Letters from Approved Homes in reply to special needs published in the Bureau should state terms and full particulars, and be sent prepaid under cover to the Editor of the Bureau with coupon and name enclosed for identification.

4. Proprietors of Homes which have not yet been entered on the List of Approved Homes, but have spare accommodation likely to suit special needs, are invited to write for an application form for registration. The fee for registration, which includes two announcements of the Home in the Bureau, is 5s.

5. Special needs of every description relating to those who are sick or in difficulties are made known in these columns **free of charge.**

6. All communications to be addressed to The Editor of The Hospital, 28 Southampton Street, Strand, London, W.C., and marked "Bureau of Information."

### Approved Homes.

Note.—*No Home is added to the List without direct medical and other testimony to its good management.*

**Bucks.**—Braeside, Horn Hill, Chalfont St. Peter. Proprietress: Miss Crighton. Epileptic and slight mental cases. Backward and delicate children. Out-door country life, gardening, driving, educational advantages.

**Cheltenham.**—St. Neots, Marle Hill Parade, Cheltenham. Proprietor: Martin E. Martin. Vibro or electric massage. Good accommodation for persons requiring rest and home comforts. Pleasant garden, with vinery. Close to spa and park.

### Nursing in the Empire and Abroad.

**Massage in Vancouver.**—Nurse-masseuse, practising in Aberdeen, is anxious to go out to Vancouver in the same capacity, and wishes for the names of hospitals in that city. Write to the Lady Superintendent, Vancouver General Hospital, and to the Matron, St. Paul's Hospital, Vancouver, enclosing postal coupon for reply. We are advised that the demand for trained nurses in British Columbia is very small, and that it is unwise to emigrate unless a promise of work be secured beforehand. The demand is chiefly for purely manual workers.—Nurse B.

**Maternity Nursing in Johannesburg or Pretoria.**—Opening wanted by maternity nurse with C.M.B. certificate and nine months' general training. The C.M.B. certificate will enable you to receive a "certificate of competence in midwifery" from the Transvaal Medical Council (fee £1), but it must be supported by a certificate of good character recently signed by a medical practitioner; a certificate of good health, signed by a medical practitioner in the Transvaal; and other formalities. You will not be eligible to practice as a nurse, and, as the supply of fully-trained nurses with midwifery certificates often exceeds the supply, we cannot advise you to go out on the chance.—V. McC.

### Training and Employment.

**Nurse "au Pair."**—The matron of a convalescent home for men on the south coast is willing to receive a helper who would make herself generally useful and nurse any patient who happened to require extra care. Good opportunity for nurse who is not strong and desires home in good climate through the winter. Prepaid letters forwarded to R. C. H.

### Needs and Helpers.

**Home for Slightly Defective Child.**—It is desired to place a little boy, aged six and a-half, backward in speech and not mentally bright, in home suited to his needs. The parents are not well off, but can pay a small sum weekly.—X. Y. (125a).

**Home for Incurables.**—In order to get a gentleman into the Home for Incurables either at Putney or Streatham, it is necessary first to get a nomination from a subscriber. The case will then be considered by the medical authorities, and if found suitable will be placed on the voting list, and you will have to issue an appeal for votes and canvass for them.—Requiem.

### Homes Wanted.

**Winter Quarters for Phthisical Patients.**—We are in correspondence with a nursing home in Italy for you, and will forward letter when received. We do not know of any suitable address in Arcachon, but perhaps some correspondents can supply one. We note that you require a sunny bed-sitting room with lavatory and bath on same floor, and good plain food in a quiet house in warm, dry climate. Have you tried Las Palmas, Grand Canary? The return fare from Liverpool for six months is £18 18s., which includes a fortnight at the hotel. Once there, you could procure pension accommodation at Monte, up in the hills, at about the terms you name, and the air has a wonderfully restorative effect in many cases. Write for particulars of voyage to Elder, Dempster, 4 St. Mary Axe, London, E.C., or to the Yeoward Line, Stanley Street, Liverpool, who afford a very cheap passage.—Emm. Der. (124a).

**Work Cure.**—Refined home, with regular occupation under trained supervision, required for active and energetic lady, aged thirty-five, in danger of nervous breakdown from private worries. The idle life inevitable at ordinary nursing homes renders them unsuitable, and the main problem is to divert attention and arouse interest in life once more. Prepaid replies, which should mention terms, forwarded to "Seeker" (123a).

### Letter-Box.

M. R.—We are much obliged for kind reply received *re* home for worker "au pair," and have asked her to write to you direct.

Australasian.—We were delighted to have your cordial appreciation of the kindness received from Miss Richardson in her Totland Bay home. It is specially pleasant to hear of hospitality extended to an "overseas" nurse.

G. H. H.—Impossible to communicate privately with D., who does not wish letters sent to address given.

Mrs. N.—Yes, we did mention your home, but the neighbourhood of London was preferred.

Sister R. Smith.—Chronic and District Nurses Required.—We are greatly indebted to you for dealing with the wants indicated by our correspondent.

H. O. B.—Nurse for Convalescent Home.—Very glad to know that the Bureau has been of service and that you have met with the nurse you require through its agency.

**A leaflet containing particulars of the Bureau will be forwarded free on application.**

### The Editor's Letter-Box.

### The Lord Mayor and "Hospital Saturday."

*To the Editor of* The Hospital.

### The Medical Exhibition.

The annual exhibition organised by the *British and Colonial Druggist* at the Horticultural Hall in Vincent Square has become an event of the year to which the doctor in London and its neighbourhood looks forward with keen anticipation of pleasure and profit. Essentially professional, limiting its appeal purely to those who have an active business interest in its exhibits, the exhibition attracts by reason of its splendid organisation, its catholicity, and its undoubted usefulness. A couple of hours spent here in leisurely strolling from stall to stall and occupying oneself in talk with those who preside over the various shows, means so much new knowledge gained, so much information amassed that invariably proves useful. Everything, this time as always before, was done to make visitors comfortable and at their ease. If possible the displays were better arranged and the various items better explained than at any former exhibition, which is saying a great deal. Where so much of interest claims attention, it is almost invidious to single out special features, but there were several novelties that may honestly be regarded as worthy of particular mention. The exhibits of surgical and hospital apparatus were excellent: we noticed fine displays by the various well-known firms. Messrs. Oppenheimer, Son and Co. showed, in addition to their variety of new drugs, the interesting Oxinhalator, a portable apparatus for the immediate and automatic production of pure oxygen. This apparatus is safe, reliable, and should prove a boon to those who cannot readily obtain the compressed gas. The Hoffmann-La Roche Company showed samples of their new Omnopon tablets, a form in which this already popular drug should win further popularity, as it considerably facilitates the administration. The well-known firm of Martindale exhibited mannitol and allied preparations that attracted considerable attention. At Wulfing's stall Formamint and the new urinary disinfectant which the firm has just put on the market were eagerly discussed. Close by R. H. Woodland and Sons had their improved invalid chairs of the well-known "Eastbourne" type, and the Endolytic Tube Company showed their improved testing-tubes, by means of which bedside urine-testing becomes a fine art reduced to kindergarten simplicity. These tubes deserve to be much better known than they appear to be; like a famous soap, once used they will always be used. Foods and food products, wines and beverages, diabetic specialities, and similar attractions were to be seen in plenty. The Skeffington invalid beds and lifters, though not exactly novelties, demanded notice by reason of their improvements and sterling quality.

The usefulness of this annual doctors' show can hardly be over estimated, and we congratulate our contemporary on the success that attends its yearly attempt to place the profession in a position to judge by contrast and comparison of the merits of the numerous novelties that clamour for recognition and trial.

